# Sleeping well

**DOI:** 10.1186/1741-7015-11-19

**Published:** 2013-01-24

**Authors:** Mithu Sen, G Bryan Young

**Affiliations:** 1 Department of Medicine, Division of Critical Care Medicine, Western University, London, Ontario, N6A 5A5, Canada

**Keywords:** vegetative state, minimally conscious state, actigraphy, circadian rhythm

## Abstract

In a study by Cruse *et al. *published in BMC Medicine, patients with severe brain damage who were in the Vegetative or Minimally Conscious States (VS or MCS, respectively) from traumatic and nontraumatic etiologies had assessments of circadian rhythms using an actigraph, a device worn on a limb to evaluate circadian rhythmicity, in this population. This is a novel approach and is being used as a surrogate for polysomnography and other reference standards. Cruse *et al. *showed more disruption in circadian rhythms in the VS when compared to the MCS. This suggests that more brain injury occurs in the areas that control circadian rhythmicity in VS than in MCS patients. The study provides opportunities for improved prognostication and rehabilitation strategies in this patient population.

## Commentary

*Sleep is the golden chain that ties health and our bodies together*. (Thomas Dekker)

The Vegetative and Minimally Conscious States (VS and MCS, respectively) are severe disorders of consciousness that reflect severe brain dysfunction/damage. Vegetative patients have preserved vegetative functions (temperature, hemodynamic and hormonal regulation and breathing) and are awake (with arousability with eye opening and sleep-wake cycles) but show no behavioral evidence of awareness (no eye contact, tracking, obeying of commands or emotional responses). Minimally conscious patients show minimal and limited but definite evidence of some aspect(s) of awareness, for example, visual tracking, movements or emotional responses triggered by relevant environmental stimuli, even very simple command-following or verbalization as isolated phenomena.

Cruse and colleagues report the results of actigraphy assessments of circadian sleep-wake rhythms in patients with VS and MCS in research published in *BMC Medicine *[1]. They studied patients with traumatic and nontraumatic brain injury at widely ranging intervals from the ictus (0 to 290 months). Actigraphy uses a watch-shaped device that is strapped to the arm and is used to measure limb movements in sleep as a surrogate for estimating the sleep-wake cycle. The actigraphs (or actimeters) can provide patterns of activity that can be used to outline circadian rhythms [2]. Four distinct aspects of movement are captured: amplitude, acrophase (the point in the sleep-wake cycle when activity is maximal), the mesor (the mean of the rhythm) and a goodness-of-fit curve to show the robustness of the circadian rhythm. This methodology has been previously validated by comparing the results with standard polysomnography [2].

The principal finding was that as a group VS patients (n = 18) had more significantly disrupted circadian rhythms than did MCS patients (n = 37).

There may be theoretical advantages and disadvantages in using actigraphy in this population instead of reference standards of objective measures, such as formal polysomnography or dim-light melatonin onset, or subjective measures, such as sleep log [3]. There was considerable heterogeneity in the anatomy and severity of the brain damage among the patients. Polysomnographic sleep staging relies primarily on the electroencephalographic (EEG) changes over time along with other variables. The EEG records rhythms from the cerebral cortex. The cortex may be somewhat dissociated from subcortical structures, namely the hypothalamus and thalamus, which drive the sleep-wake cycle, especially in cases of severe head injury with diffuse axonal injury. Thus, actigraphy, which reliably measures the motor output of various sleep and wakefulness stages, may have an advantage. On the other hand, if the motor pathways are damaged, this may dissociate motor output from activity of the deep grey structures.

There were confounders present, especially the use of drugs that could alter the EEG, sleep-wake cycle including sleep staging, but at least these were well documented.

The findings of Cruse *et al. *[1] are of both theoretical and practical interest. It makes intuitive sense that circadian rhythms would be more disrupted in patients with VS than MCS as the former state reflects a greater degree of brain damage than the latter. As the authors mention, the suprachiasmatic nucleus in the thalamus (the 'pacemaker' for the circadian rhythm) need not be directly damaged to produce circadian disruption, but is driven to some extent by other structures, especially the thalamus [4], which is commonly damaged directly or through transsynaptic degeneration [5]. It follows that the degree of circadian rhythm disruption as measured by actigraphy, in this novel application, may have prognostic significance. The other point, which seems to be making itself more apparent with advances in technology, is that the clinical evaluation in severely brain-damaged patients is often inaccurate or misleading [6,7]. Simple eye-opening to differentiate wakefulness from sleep is not always reliable.

There are some exciting prospects for extending this type of research. Combining polysomnography with actigraphy may address the variable pathologies in brain-injured patients and allow better characterization and validation of sleep compared to wakefulness. It would also be of value to study patients serially from the time of ICU admission onwards to note when true sleep-wake circadian cycles return and to correlate these with other measures of structure and function to arrive at an earlier prognosis. This may also help in the selection of patients who could be fast-tracked for early rehabilitation, which has been shown to improve cognitive and discharge destination outcomes [8].

## Abbreviations

EEG: electroencephalographic; MCS: Minimally Conscious State; VS: Vegetative State.

## Competing interests

The authors declare that they have no competing interests.

## Authors' contributions

MS and GBY drafted the editorial and have read and approved the final manuscript.

## Authors' information

MS is an Associate Professor in the Department of Medicine, Divisions of Critical Care Medicine and Respirology/ Sleep Medicine and Schulich School of Medicine and Dentistry, Western University, London, ON, Canada. She is certified in sleep medicine and is a Fellow of the American Academy of Sleep Medicine (FAASM) as well as board certified in Critical Care and Pulmonology in both Canada and the US. She is the Director of Education for the Royal College of Physicians and Surgeons of Canada (RCPSC) Postgraduate Residency Training Program for Adult Critical Care, Western University. GBY is a Professor in Clinical Neurological Sciences specializing in neurocritical care.

## Pre-publication history

The pre-publication history for this paper can be accessed here:

http://www.biomedcentral.com/1741-7015/11/19/prepub
